# Intracoronary Delivery of Human Mesenchymal/Stromal Stem Cells: Insights from Coronary Microcirculation Invasive Assessment in a Swine Model

**DOI:** 10.1371/journal.pone.0139870

**Published:** 2015-10-19

**Authors:** António Fiarresga, Márcia F. Mata, Sandra Cavaco-Gonçalves, Mafalda Selas, Irina N. Simões, Eunice Oliveira, Belmira Carrapiço, Nuno Cardim, Joaquim M. S. Cabral, Rui Cruz Ferreira, Cláudia L. da Silva

**Affiliations:** 1 Cardiology Department, Hospital de Santa Marta, Lisboa, Portugal; 2 Nova Medical School, Universidade Nova de Lisboa, Lisboa, Portugal; 3 Department of Bioengineering and iBB-Institute for Bioengineering and Biosciences, Instituto Superior Técnico, Universidade de Lisboa, Lisboa, Portugal; 4 National Institute for Agrarian and Veterinary Research, Oeiras, Portugal; 5 Faculty of Veterinary Medicine, Universidade de Lisboa, Lisboa, Portugal; Indiana University School of Medicine, UNITED STATES

## Abstract

**Background:**

Mesenchymal stem/stromal cells have unique properties favorable to their use in clinical practice and have been studied for cardiac repair. However, these cells are larger than coronary microvessels and there is controversy about the risk of embolization and microinfarctions, which could jeopardize the safety and efficacy of intracoronary route for their delivery. The index of microcirculatory resistance (IMR) is an invasive method for quantitatively assessing the coronary microcirculation status.

**Objectives:**

To examine heart microcirculation after intracoronary injection of mesenchymal stem/stromal cells with the index of microcirculatory resistance.

**Methods:**

Healthy swine were randomized to receive by intracoronary route either 30x10^6^ MSC or the same solution with no cells (1% human albumin/PBS) (placebo). Blinded operators took coronary pressure and flow measurements, prior to intracoronary infusion and at 5 and 30 minutes post-delivery. Coronary flow reserve (CFR) and the IMR were compared between groups.

**Results:**

CFR and IMR were done with a variance within the 3 transit time measurements of 6% at rest and 11% at maximal hyperemia. After intracoronary infusion there were no significant differences in CFR. The IMR was significantly higher in MSC-injected animals (at 30 minutes, 14.2U *vs*. 8.8U, p = 0.02) and intragroup analysis showed a significant increase of 112% from baseline to 30 minutes after cell infusion, although no electrocardiographic changes or clinical deterioration were noted.

**Conclusion:**

Overall, this study provides definitive evidence of microcirculatory disruption upon intracoronary administration of mesenchymal stem/stromal cells, in a large animal model closely resembling human cardiac physiology, function and anatomy.

## Introduction

Cardiovascular diseases represent the leading cause of death in developed countries despite major advances in its treatment and prevention, and these are also a major cause of disability, lost productivity and increased health costs worldwide [1]. Most cardiovascular diseases are associated with loss of functional cardiomyocytes, which are not replaced due to the limited regenerative capacity intrinsic to the heart. In recent years, cell-based therapy has been presented as a potential therapeutic strategy for cardiac regeneration [2].

Mesenchymal stem/stromal cells (MSC), in particular, have emerged as a promising candidate for cell-based therapies [3]. These cells have important intrinsic features for therapeutic settings namely: easy isolation from a small aspirate of bone marrow, adipose tissue or from other sources of perinatal origin (*e*.*g*. umbilical cord) and high *in vitro* expansion potential [4,5], multilineage differentiation capacity [6], ability to modulate immune responses and secrete various cytokines that play a role in processes such as angiogenesis, inflammation, cellular migration and apoptosis [7]. Although there is no clear evidence that MSC can differentiate into myocardial cells [8], the advantages of a MSC-based therapy could lie in the immunomodulatory effects mediated by these cells, as well as the ability of MSC to promote growth, survival or differentiation of other cells in the damaged myocardial area through paracrine mechanisms [9]. These properties have encouraged pre-clinical and clinical research to explore the potential role of MSC as a cell-based treatment for heart disease [3].

The use of intracoronary (IC) artery infusion in cell-based therapy is based on standard knowledge and catheterization techniques allowing direct delivery of a large number of cells into myocardial regions. With this advantage, the IC route has been the most used delivery method of hematopoietic cells (*i*.*e*. mononucleated cells, MNC), including stem/progenitor cells, in cardiovascular cell-based research [2]. However, its use for administration of MSC is still a matter for debate. With a diameter of 10–20 μm, MSC are larger than heart capillaries (5–10 μm) and there is the risk of microcirculation obstruction [10]. The potential safety issues of intracoronary MSC-based therapies, namely microinfarctions, should be addressed with solid pre-clinical research, as current data is limited and controversial.

Coronary flow reserve (CFR) is the maximum increase in blood flow through the coronary arteries above the normal resting volume and is measured to assess the flow in the epicardial artery and in the microcirculatory bed. The index of microcirculation resistance (IMR), allows a quantitative, invasive, and real-time evaluation of the coronary microcirculation status independently of the epicardial vessel. IMR is defined as distal coronary pressure multiplied by the hyperemic mean transit time (mm Hg · seconds, or units [U]) [11]. Current interventional cardiology techniques allow for the simultaneous measurement of CFR and IMR with a pressure-temperature sensor-tipped wire. Mean transit time is derived from thermodilution after IC injection of room-temperature saline and it correlates to the coronary absolute flow. Disruption of the microvascular bed is associated with a significant increase in IMR and this can be determined immediately after an IC intervention using the same catheter based procedure. IMR assessment is expected to clarify how MSC IC infusion affects heart microcirculation.

## Objectives

The overall goal of the present work was to study the IC artery delivery of human MSC in a large animal model—swine—closely resembling coronary circulation and arterial anatomy in humans. Specifically, this study was designed to examine heart microcirculation damage with IMR assessment after MSC IC injection in the animals compared to a control group (*i*.*e*. no cells administered). We also evaluated the differences in hemodynamic, electrocardiographic and coronary epicardial artery flow parameters upon administration of MSC.

## Materials and Methods

### Overview

Eighteen swine (males Duroc and females F1 Large White X Landrace) with a weight of 25 to 40 Kg (31 ± 5 Kg) were randomized to receive either (i) 1 million MSC/kg corresponding to approximately 30x10^6^ MSC resuspended in phosphate buffered saline (PBS) supplemented with human albumin (HA), or (ii) cell-free PBS/HA solution (placebo—control group). All procedures were the same between the two groups. The operators performing all measurements were blinded to which group the animal belonged to.

### MSC preparation

MSC used in this study were part of the cell bank available at the Stem Cell Bioengineering and Regenerative Medicine Laboratory, Instituto Superior Técnico (IST-iBB). These cells were isolated from Bone marrow (BM) aspirates from healthy donors upon informed consent according to the protocol described by dos Santos and co-workers [12]. Briefly, the mononuclear fraction was separated using a Ficoll gradient and cells were isolated based on their ability to adhere to tissue culture plastic (polystyrene) upon cultivation in low-glucose Dulbecco’s modified Eagle’s medium (DMEM) (Gibco) supplemented (10%) with MSC-qualified fetal bovine serum (FBS) (Hyclone). For cell preparation in the present study, multiple vials of BM MSC from a single donor (kept cryopreserved in a liquid/vapour phase nitrogen tank) were thawed and plated at 3000–6000 cells/cm^2^ on T-175 flasks (BD Falcon) using DMEM-10%FBS and expanded upon consecutive passaging to reach the target cell doses for administration. Cells at passages P5-P7 were used in the present study. Cell number and viability were assessed using the Trypan Blue exclusion method (Gibco) and counting cells on a hemocytometer under an optical microscope (Olympus).

MSC were characterized by flow cytometry using specific monoclonal antibodies according to criteria defined in the literature [13]. Over 95% of the population should express CD73, CD90 and CD105 and not express (less than 2%) CD14, CD19, CD31, CD34, CD45, CD80 and HLA-DR. For this purpose, cells were incubated for 15 minutes in the dark with a panel of mouse anti-human fluorescent-labeled monoclonal antibodies (all from BioLegend, except anti-CD105 (Invitrogen)). Cells were then washed with PBS and analyzed by flow cytometry. A minimum of 10 000 events were collected for each sample and the CellQuest™ software (Becton Dickinson) was used for acquisition and analysis. The differentiation potential of MSC was tested using biochemical methods after *in vitro* induction of osteogenesis, adipogenesis, and chondrogenesis using specific culture media.

In order to prepare administration, expanded cells were resuspended in a HA-containing (1%) solution prepared by dilution (1:30) of a stock solution (30%, Albunorm, Octapharma) in PBS. Multiple samples of cell suspensions were observed under optical microscopy at different time points upon resuspension to verify the occurrence of cell aggregation. Cells were transported from the cell culture laboratory at IST-iBB to animal facilities at room temperature and administered within 2 hours.

#### Animal care and catheterization procedures

Animal procedures were approved by the Ethical and Animal Welfare commission of the Veterinary Medical School, Universidade de Lisboa, and the study was authorized by the national regulatory authorities. All handling and care followed the European directive 2010/63/EU on the protection of animals used for scientific purposes.

Animals fasted overnight prior to the experiments. Anesthesia was induced by intramuscular injection of azaperone (2 mg/kg), atropine (1 mg) and after 15 minutes, ketamine (20 mg/kg). In the catheterization laboratory, a venous catheter (22 G) was placed in the marginal ear vein, through which sodium thiopental (6 mg/kg) was injected. Anesthesia was maintained with mechanical tracheal ventilation (tidal volume of 10 ml/kg) and inhaled isoflurane (2%). After placing a 6F sheath in the femoral artery, anticoagulation was achieved with intravenous heparin (300 U/ kg). Hemodynamic and electrocardiographic monitoring was maintained during the entire experiment. Aorta and left ventricle pressure measurements were done with a 6F pigtail catheter and for selective catheterization of the left main artery a 6F Amplatz left 0.75 was used. All heart catheterization procedures were conducted under fluoroscopy.

At the end of the experiment, animals that received MSC were euthanized with intravenous sodium thiopental. Control animals were treated with intravenous carprofen (2 mg/kg) plus amoxicillin (15mg/kg) and kept in 48 hours quarantine with no signs of suffering.

### MSC delivery

After selective catheterization of the left main artery, a microcatheter FineCross™ (Terumo Interventional Systems) was placed in the left anterior descending coronary artery (LAD), just distal to the first diagonal branch and with the help of a .014 guiding wire BMW^®^ (Abbott Vascular). Through its central lumen, the 20 cc solution was injected at a 4 cc / min constant flow, using an oscillating infusion pump syringe to prevent cell aggregation. At the end, the microcatheter was removed and a coronariography was performed to exclude dissections.

### Coronary pressure and flow measurements

Measurements were taken prior to IC infusion and at 5 and 30 minutes post-delivery. A coronary pressure wire, PressureWire™ Certus (St. Jude Medical), was calibrated and advanced to the distal LAD, with the sensor located in the middle third of the coronary. The position of the wire was maintained during the experiment.

A RadiAnalyzer™ Xpress interface and software (Radi Medical Systems, St. Jude Medical) were used for pressure and mean transit time records. Thermodilution-derived mean transit time was assessed after a rapid injection of 3 ml of room temperature saline (*i*.*e*. PBS/HA) through the guiding catheter. Measurements were taken at resting and after maximal hyperemia induced with 10 mg IC papaverine. Three measurements were considered and the mean vales were used for analysis. Distal coronary pressure was simultaneously recorded.

**Fig 1:**
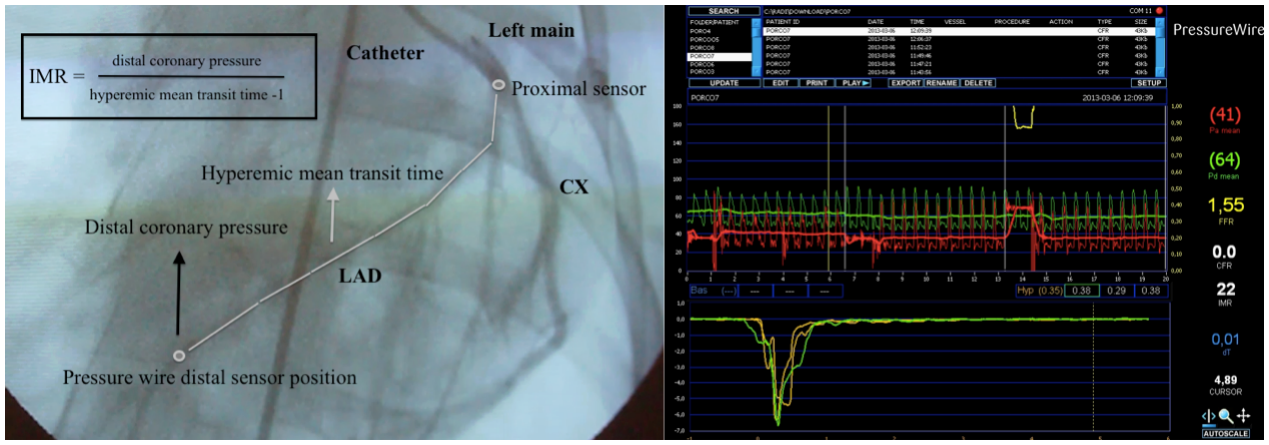
Left coronary angiography and RadiAnalyzer™ Xpress interface display. The mean transit time was estimated after a rapid IC injection of room temperature saline through the guiding catheter. Temperature change is first detected at the proximal sensor, and then saline circulates in coronary artery and reaches the distal sensor. The distal sensor of the pressure wire also assesses de distal pressure. IMR is calculated with these two variables. Thermodilution curves could be seen at the bottom of the interface display (green and yellow). In the middle, aortic pressure (red curve) and distal coronary pressure (green curve) are also shown. LAD: left anterior descendent artery; CX: circumflex artery, IMR: index of microcirculatory resistance.

CFR was defined as resting mean transit time divided by hyperemic mean transit time. IMR was calculated distal coronary pressure multiplied by the hyperemic mean transit time (mm Hg · seconds, or units [U]) (Fig 1).

### Electrocardiographic, hemodynamic, and angiographic evaluation

Twelve-lead electrocardiograms (ECG) were done before heart catheterization and 30 minutes after IC delivery of MSC or cell-free solution. Electrocardiographic monitoring was maintained through the experiment. Heart rate, ventricular arrhythmias and ECG profile changes with evidence of acute myocardial ischemia were assessed.

Intraventricular and aortic pressures were recorded at the beginning of the experiment and 30 minutes after IC infusion.

After selective catheterization of the left main artery, 0.5 mg of isossorbide dinitrate was injected for coronary dilation. Left coronary angiograms were done with manual injection of 10 cc of iodine-based contrast media prior to IC infusion, immediately after and at 30 minutes. Coronary epicardial flow was classified with the TIMI scoring system.

### Statistical analysis

Data are presented as mean ± standard deviation. Normality was assessed using the Shapiro–Wilk’s tests. Comparisons were made among control and MSC animal groups using Student's *t*-test for independent variables, significance was set at the 0.05 level. Intragroup analysis was done using repeated measures ANOVA model with post hoc paired t-tests with Bonferroni's correction, and a corrected p value < 0.05 was taken as significant. Statistical analysis was performed using site-licensed SPSS^®^ statistical software.

## Results

**Fig 2:**
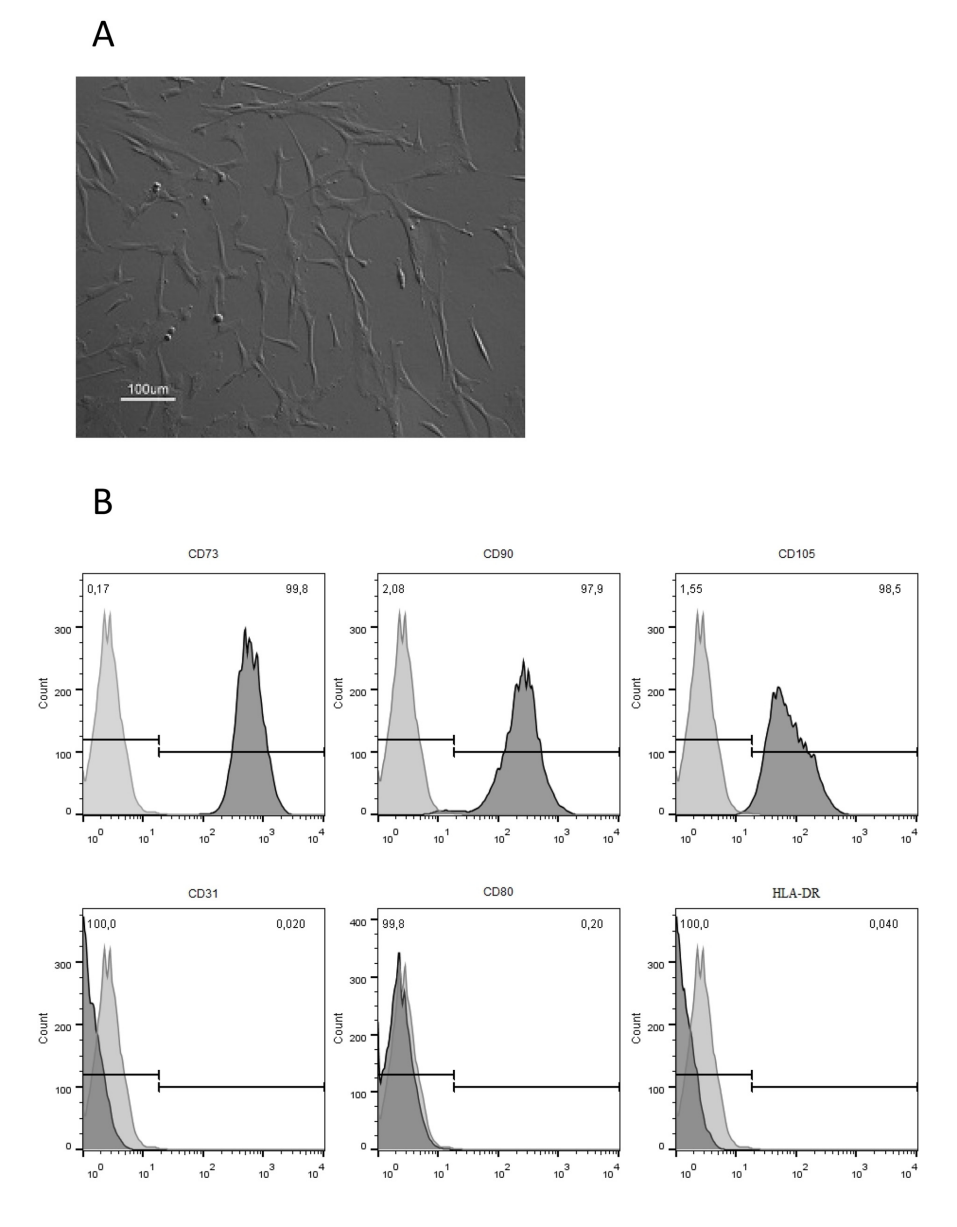
Characterization of bone marrow (BM)-derived mesenchymal stem/stromal cells (MSC) expanded on tissue culture flasks using DMEM culture medium supplemented with 10% FBS. (A) Morphology of cultured cells assessed by optical microscopy. (B) Immunophenotype of cultured MSC assessed by flow cytometry according to Materials and Methods description. Percentage (%) displayed in the upper right corner of each histogram indicates the expression of each antigen.

For the purpose of this study, BM-derived MSC administered into the animals were previously expanded onto tissue culture flasks through consecutive passaging and maintained their characteristic spindle shape morphology (Fig 2A), immunophenotype (Fig 2B), as well as their multilineage differentiation ability (data not shown).

Hemodynamic parameters are presented in Table 1. There were no significant differences in baseline parameters between MSC and control groups. After MSC injection there were no significant changes in heart rate, left ventricular end-diastolic pressure and systolic aortic pressure. There was no significant coronary artery narrowing and all animals had TIMI 3 grade distal blood flow immediately after and at 30 minutes of IC infusion. During the procedure no ST-segment elevation or ventricular arrhythmias were observed.

**Table 1:** Weight, baseline and post-delivery hemodynamic parameters in *Control* and *MSC* animals. Means and standard deviation, differences assessed with Student's *t*-test for independent variables (n = 9).

	CONTROL	MSC	P
Weight (kg)	31 ± 2	32 ± 2	0.8
Heart rate (bpm)	106 ± 5	100 ± 5	0.5
Aortic systolic pressure (mmHg)	98 ± 7	93± 3	0.4
End-diastolic LV^1^ pressure (mmHg)	7.7 ± 2	7.3 ± 2	0.7
Post-delivery heart rate (bpm)	114 ± 23	111 ± 15	0.7
Post-delivery aortic systolic pressure (mmHg)	96 ± 18	88± 13	0.3
Post-delivery end-diastolic LV^1^ pressure (mmHg)	8.3 ± 3	8.2 ± 3	0.9

^1^Left ventricle.

The variance within the 3 hyperemic transit time measurements was 6% at rest and 11% at maximal hyperemia in the studied population (18 animals divided into two groups, n = 9). The mean reduction in distal coronary pressure after IC papaverine was 18 ± 7.0 mmHg in control animals and 14 ± 5.0 in MSC animals (p = 0.2), a relative decrease of 27% and 22%, respectively.

The mean values of CFR and IMR are presented in Table 2. CFR and IMR were similar between the 2 groups at baseline. After IC infusion there were no significant differences in CFR, although a small decrease was observed in the MSC group. Concerning IMR, no significant differences were observed at 5 minutes, but a significant increase was observed at 30 minutes in the MSC-injected animals (p = 0.02).

**Table 2:** Coronary flow reserve and index of microcirculatory resistance in *Control* and *MSC* animals. Means and standard deviation, differences assessed with Student's *t*-test for independent variables (n = 9).

	CONTROL	MSC	P
Baseline CFR^1^	3.8 ± 1	4 ± 2	0.7
Baseline IMR^2^ (U)	8.1 ± 1	6.7 ± 0.6	0.3
Post-delivery CFR^1^—5 min.	3.6 ± 1	3 ± 2	0.5
Post-delivery CFR^1^—30 min.	3.8 ± 2	2.3 ± 1.5	0.06
Post-delivery IMR^2^—5 min. (U)	9.8 ± 1	15.3 ± 2.8	0.08
Post-delivery IMR^2^—30 min. (U)	8.8 ± 1	14.2 ± 1.8	0.02

^**1**^ Coronary flow reserve

^2^ Index of microcirculatory resistance.

**Fig 3:**
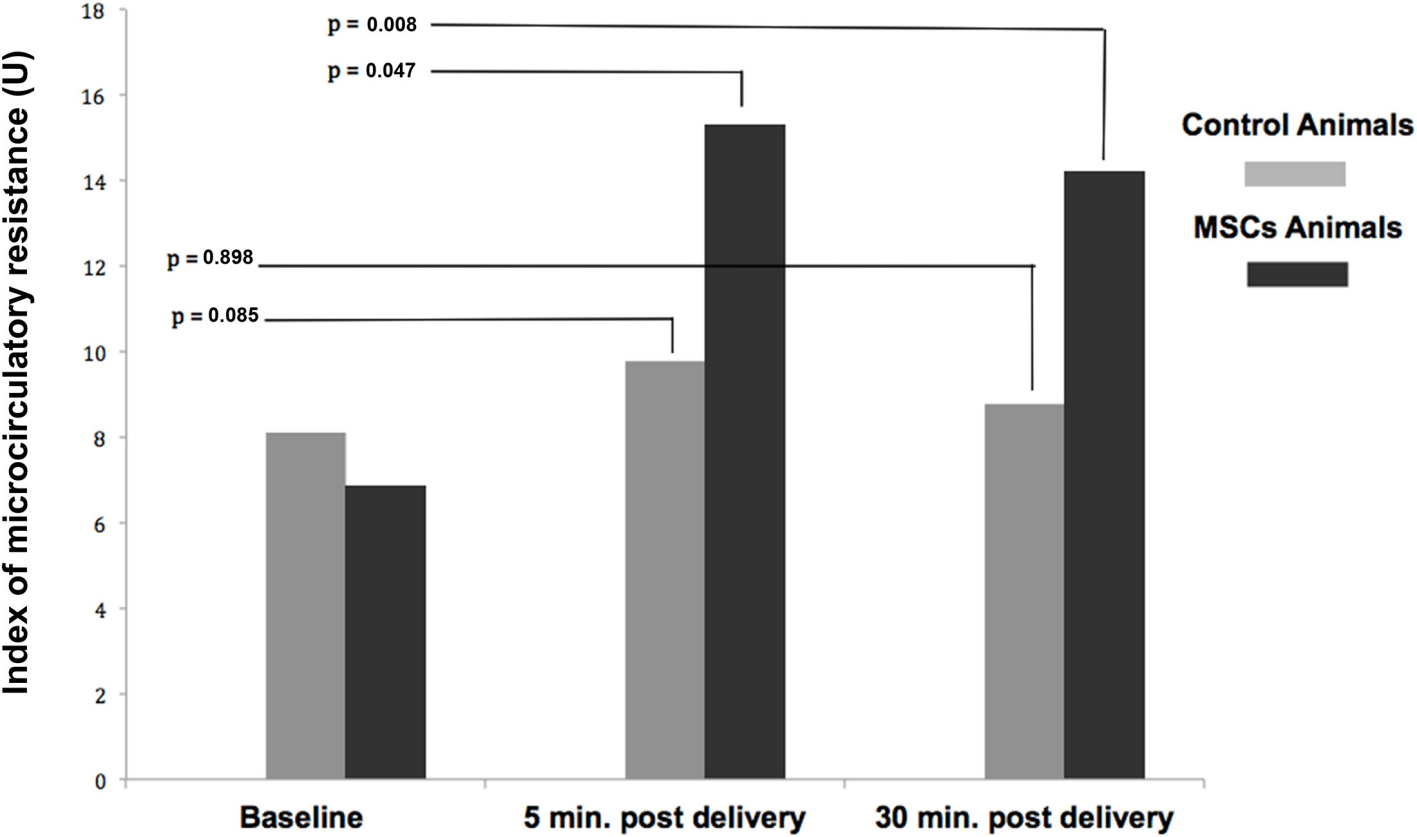
The index of microcirculatory resistance—results at baseline, 5 and 30 minutes. Baseline, 5 and 30 minutes post delivery IMR values are shown for Control and MSC group (n = 9). Differences from baseline were assessed with repeated measures ANOVA and P-values are adjusted for multiple comparisons (Bonferroni).

Fig 3 shows the results of IMR assessed at 5 and 30 minutes after intracoronary infusion. In repeated measures ANOVA mean IMR differed statistically significantly between time points in animals that received cells (*F* (2–16) = 8.925, p = 0.002). In these animals the baseline IMR was 6.7 ± 0.6 U, and after IC infusion increased to 15.3 ± 2.8 U at 5 minutes (corresponding to a 128% increase, p = 0.047) and to 14.2 ± 1.8 U at 30 minutes (112% increase, p = 0.008). In the control group there was a barely detectable statistically significant difference in mean IMR (*F* (2–16) = 3.897, p = 0.042), and the Bonferroni post hoc test did not reveal significant difference between time points. The baseline IMR values in control animals was 8.1 ± 1.0 U, and after IC infusion increased to 9.8 ± 1.0 U at 5 minutes (corresponding to a 21% increase, p = 0.085) and to 8.8 ± 1 U at 30 minutes (9% increase, p = 0.898). All relevant data are presented in the supporting information (S1 File).

## Discussion

After several years of cardiovascular cell-based research, the optimal cell type for regenerating the damaged myocardium and trigger neovascularization and angiogenesis, as well as the best cell delivery route, have not been established yet.

MSC in particular have been extensively investigated in cardiac regenerative therapy trials during the past decade [14,15]. In pre-clinical studies these cells have been shown to engraft in the heart, reduce infarction size and allow for an improvement in ventricle contractile function in rodent [16,17] and larger animal models [18,19].

In the present work, we studied the IC delivery of human BM-derived MSC in a swine model, closely resembling coronary circulation and arterial anatomy in humans. We chose to use culture-expanded, human BM-derived cells defined by the standards of the International Society of Cellular Therapy (ISCT) [13], since these cells, despite representing a heterogeneous cell population, have been widely exploited experimentally and in clinical trials. For instances, Hare and co-workers reported in 2009 a double-blind controlled phase I study demonstrating the safety of the intravenous route of MSC in patients with myocardial infarction [20]. More recently, Bartunek and colleagues, in the C-Cure trial, demonstrated the safety associated with signs of clinical benefit of MSC endomyocardial injections in patients with chronic heart failure [21].

The IC cell delivery of MSC has proven to result in an increased cell engraftment within the infarcted tissue of porcine compared to the intravenous route [22]. The comparison of IC delivery *versus* the endocardial route is more controversial [22,23]. While IC delivery is based on coronary procedures that are part of clinical practice nowadays, the endocardial administration of cells relies on more complex techniques that interventional cardiologists are not familiar with. Nevertheless, despite the advantages of the IC route, there is apprehension about the size of MSC and the possibility of microvascular obstruction [24,25], myocardial microinfarctions [26], and decreased coronary flow [21] upon MSC delivery. In this context, preclinical data has been controversial but some recent studies support the safety and clinical benefit of MSC IC delivery [27,28,29].

It has been hypothesized that microcirculatory entrapment of MSC and their consequences to coronary flow are probably dose related. For instance, Hong and colleagues performed an IC dose escalating study in 3 pigs, injecting 1, 3, 10, 30, 100x10^6^ of adipose-derived stem cells with 30 minute intervals [30]. Coronary blood was not changed at a cumulative dose of 14x10^6^, but further injections after cumulative dose of 44 x 10^6^ were associated with a progressive decrease in the coronary filling time culminating in hypokinesia of the anterior apical wall. With this data in mind, we chose to use infusions of 30x10^6^ MSC (roughly corresponding to 1x10^6^ MSC/kg) in the present study. This could be a suitable dose since there were no periprocedural complications with IC infusion of MSC and no hemodynamic deterioration, arrhythmias or compromised coronary epicardial vessel flow were observed.

Currently no IC delivery strategy has emerged as the optimal administration method for cell administration. Previous studies have used the stop-flow methodology as described by Strauer and co-workers [31], with slight variations between these. Briefly, with this technique a coronary vessel is occluded with an over-the-wire balloon and cells are injected through the central lumen of the balloon-catheter in several occlusion-reperfusion series. Theoretically this could avert cell backflow or rapid cell washout. On the other hand, this technique may lead to MSC accumulation, and even aggregation, increasing the risk of cell entrapping. Another disadvantage is the risk of coronary dissection with the balloon, particularly in non-stented vessels. In our experiment, human MSC were slowly infused using a microcatheter and an infusion pump syringe, at a constant flow and without coronary occlusion. We speculate that with this approach MSC are drifted towards the microcirculation in smaller numbers and at a constant rate, which potentially prevents or minimizes microvessel sludging. Our findings support that this continuous infusion with coronary flow maintained could potentially be a safer way of IC administration of MSC.

To our best knowledge, the present study is the first one to address the acute microcirculatory effects of IC MSC delivery using IMR measurements and a controlled and blind research design. IMR evaluation relies in a complex technique and our group previously performed its validation in a porcine model [32].

Here, MSC infusion resulted in a significant, but not so impressive, increase of IMR to 15.3 ± 3 U. This suggests that several degrees of microcirculation compromise could be distinguished by IMR assessment, which could be useful towards the establishment of an optimal MSC dose scheme to study safety and efficacy. We believe that demonstrating changes in microcirculation, in vivo and in real time, after the intracoronary administration of these cells is not trivial, since we still lack the limits on what is normal or what defines an unwanted effect of any intervention. However, it can be the first step to clarify the potential clinical value of IMR in future studies.

We also observed a small increase in IMR at 5 minutes in control animals (*i*.*e*. no cells administered) that could be related to coronary manipulation during saline infusion. In fact, only after 30 minutes, a significant difference between groups occurs. This possibly means that we should wait before measuring IMR instead of doing it immediately after an intervention to assess acute effects of any procedure.

Hong and collaborators also addressed the microcirculatory effects of IC injections of adipose-derived stem cells with IMR measurements [30]. These authors reported a significant increase from baseline in IMR at 7 days in the high-dose group (receiving 50x10^6^ cells) (11 ± 1.3 *vs*. 17.8 ± 3.2, p = 0.04, respectively), and no significant changes in the low-dose group (10x10^6^ cells). Despite the different MSC source (adipose) used and a distinct IC infusion technique (*i*.*e*. cells were delivered at a rate of 1.3 ml/min with alternating 3-min infusions and 3-min periods of reperfusion), our results are in line with those obtained by Hong and co-workers, which clearly demonstrates the potential for microvasculature obstruction with MSC IC delivery. The differences observed between the two studies, namely the more expressive increase in IMR observed in our work might be also the result of differences in the studied population (healthy *vs*. post-myocardial infarction animals), and time of IMR assessment. In contrast to the previous work, our operators were blinded for the assessment of IMR, which avoids operator-induced bias.

Most importantly, CFR had no significant change, confirming that epicardial circulation was unaffected during the experiment and that IMR is a better and more specific parameter for microcirculation assessment.

## Study Limitations

We used healthy animals with normal microcirculation and extrapolating our results to the context of heart disease should be done with caution. A histological study to confirm the presence of microvessel obstruction was not performed, and so we can only consider this as a hypothesis for the occurrence an increase in IMR. On the other hand, a side-by-side comparison between IC infusion techniques was not done and thus our results warrant further research.

Overall, IMR enables the assessment of the microcirculation status by Interventional Cardiologists. Correlating IMR values after IC delivery of MSC with data about cell fate and distribution, histological analysis and long term clinical benefit in the context of heart disease are important issues that should be addressed in future work.

## Conclusion

We demonstrate the feasibility of use IC delivery to administer culture-expanded human MSC without compromising hemodynamic, electrical stability and epicardial coronary flow. However, our study does provide definitive evidence of microcirculatory disruption exposed by a significant increase in IMR. The impact of these microcirculatory effects and the potential applications of IMR warrant further investigation.

## Supporting Information

S1 File Tables with all the relevant research results. (ZIP)
