# Supplementary material for: Intracoronary Delivery of Human Mesenchymal/Stromal Stem Cells: Insights from Coronary Microcirculation Invasive Assessment in a Swine Model
Source: PLoS One. 2015 Oct 19;10(10):e0139870. doi: 10.1371/journal.pone.0139870 (PMC4610677; doi:10.1371/journal.pone.0139870)
Supplement: S1 File — (ZIP) [file pone.0139870.s001.zip › DATA_SET.docx]

| Cells | Animal | Weight | Sex | HeartRate | BloodPressure Systolic/dyastolic/mean | LeftVentricle Telediastolic Pressure |  | Final BloodPressure | | FinalLeftVentricle Telediastolic Pressure | | Final Heart Rate | |
| --- | --- | --- | --- | --- | --- | --- | --- | --- | --- | --- | --- | --- | --- |
| 1 | 1 | 31,5 | M | 92 | 88/40/60 | 10 |  | 71/43/56 | 8 | | 120 | |  |
| 0 | 2 | 26 | M | 113 | 89/56/70 | 8 |  | 90/45/67 | 6 | | 121 | |  |
| 1 | 3 | 42 | F | 107 | 96/61/72 | 6 |  | 74/47/56 | 4 | | 84 | |  |
| 0 | 4 | 26 | M | 98 | 88/52/72 | 4 |  | 72/37/54 | 12 | | 100 | |  |
| 0 | 5 | 23 | F | 97 | 82/47/65 | 10 |  | 110/80/95 | 12 | | 129 | |  |
| 1 | 6 | 33 | F | 72 | 85/55/70 | 6 |  | 98/52/71 | 8 | | 129 | |  |
| 0 | 7 | 31,5 | F | 107 | 93/58/72 | 8 |  | 90/55/75 | 5 | | 107 | |  |
| 0 | 8 | 27,5 | F | 99 | 92/50/71 | 4 |  | 85/45/61 | 4 | | 128 | |  |
| 1 | 9 | 27 | M | 123 | 85/49/65 | 8 |  | 75/25/40 | 10 | | 115 | |  |
| 1 | 10 | 30 | M | 97 | 97/47/59 | 8 |  | 99/44/66 | 6 | | 113 | |  |
| 1 | 11 | 28 | M | 89 | 91/53/70 | 6 |  | 99/66/84 | 8 | | 101 | |  |
| 1 | 12 | 28,5 | M | 111 | 84/40/56 | 6 |  | 85/55/63 | 12 | | 94 | |  |
| 1 | 13 | 35 | M | 116 | 98/45/67 | 10 |  | 89/53/69 | 12 | | 116 | |  |
| 0 | 14 | 42 | M | 115 | 140/65/85 | 10 |  | 135/60/85 | 12 | | 157 | |  |
| 0 | 15 | 32,5 | M | 79 | 95/54/72 | 7 |  | 85/60/73 | 8 | | 82 | |  |
| 1 | 16 | 29 | F | 96 | 110/65/83 | 6 |  | 102/55/74 | 6 | | 125 | |  |
| 0 | 17 | 38,5 | F | 113 | 105/75/92 | 8 |  | 95/60/84 | 6 | | 93 | |  |
| 0 | 18 | 29,5 | F | 129 | 95/59/76 | 10 |  | 100/55/72 | 10 | | 111 | |  |

**TABLE 1 – Hemodynamic results – baseline and final.**

Cells – 0 = Control animals, 1 = MSC animals.

| Cells | Animal | Baseline Dp | Baseline Tm 1/3 | Baseline Tm 2/3 | Baseline Tm 3/3 | T Baseline Tm Mean | Pd/Tm Baseline | Baseline Dp - Hy | | Baseline Tm- Hy 1/3 | | Baseline Tm- Hy 2/3 | | Baseline Tm- Hy 3/3 | | Baseline  IMR | | Baseline  CRF | |
| --- | --- | --- | --- | --- | --- | --- | --- | --- | --- | --- | --- | --- | --- | --- | --- | --- | --- | --- | --- |
| 1 | 1 | 62 | 0,66 | 0,7 | 0,65 | 0,67 | 41 | 41 | 0,2 | | 0,23 | | 0,16 | | 8 | | 3,406779661 | |  |
| 0 | 2 | 76 | 0,48 | 0,48 | 0,49 | 0,483333333 | 36 | 42 | 0,13 | | 0,12 | | 0,15 | | 5 | | 3,625 | |  |
| 1 | 3 | 53 | 0,86 | 0,84 | 0,91 | 0,87 | 46 | 40 | 0,22 | | 0,28 | | 0,29 | | 10 | | 3,303797468 | |  |
| 0 | 4 | 67 | 0,96 | 0,92 | 0,94 | 0,94 | 62 | 44 | 0,33 | | 0,28 | | 0,33 | | 14 | | 3 | |  |
| 0 | 5 | 85 | 0,67 | 0,69 | 0,69 | 0,683333333 | 58 | 60 | 0,16 | | 0,18 | | 0,12 | | 10 | | 4,456521739 | |  |
| 1 | 6 | 58 | 0,61 | 0,73 | 0,68 | 0,673333333 | 39 | 37 | 0,18 | | 0,12 | | 0,13 | | 5 | | 4,697674419 | |  |
| 0 | 7 | 61 | 0,55 | 0,55 | 0,54 | 0,546666667 | 34 | 47 | 0,12 | | 0,14 | | 0,12 | | 6 | | 4,315789474 | |  |
| 0 | 8 | 60 | 0,62 | 0,64 | 0,55 | 0,603333333 | 37 | 29 | 0,17 | | 0,15 | | 0,21 | | 5 | | 3,41509434 | |  |
| 1 | 9 | 58 | 0,41 | 0,39 | 0,37 | 0,39 | 23 | 47 | 0,12 | | 0,11 | | 0,16 | | 6 | | 3 | |  |
| 1 | 10 | 55 | 0,69 | 0,67 | 0,64 | 0,666666667 | 39 | 36 | 0,17 | | 0,12 | | 0,14 | | 5 | | 4,651162791 | |  |
| 1 | 11 | 75 | 0,28 | 0,31 | 0,26 | 0,283333333 | 21 | 59 | 0,14 | | 0,15 | | 0,21 | | 9 | | 1,7 | |  |
| 1 | 12 | 52 | 0,72 | 0,75 | 0,71 | 0,726666667 | 39 | 39 | 0,14 | | 0,15 | | 0,14 | | 5 | | 5,069767442 | |  |
| 1 | 13 | 51 | 0,59 | 0,57 | 0,58 | 0,58 | 30 | 33 | 0,17 | | 0,18 | | 0,22 | | 6 | | 3,052631579 | |  |
| 0 | 14 | 75 | 0,47 | 0,48 | 0,49 | 0,48 | 36 | 64 | 0,14 | | 0,16 | | 0,11 | | 9 | | 3,512195122 | |  |
| 0 | 15 | 66 | 0,61 | 0,62 | 0,67 | 0,633333333 | 42 | 44 | 0,16 | | 0,11 | | 0,17 | | 7 | | 4,318181818 | |  |
| 1 | 16 | 72 | 0,7 | 0,7 | 0,69 | 0,696666667 | 50 | 55 | 0,13 | | 0,14 | | 0,16 | | 8 | | 4,860465116 | |  |
| 0 | 17 | 83 | 0,69 | 0,62 | 0,55 | 0,62 |  | 59 | 0,14 | | 0,14 | | 0,21 | | 9 | | 3,795918367 | |  |
| 0 | 18 | 77 | 0,7 | 0,69 | 0,63 | 0,673333333 | 52 | 43 | 0,19 | | 0,19 | | 0,2 | | 8 | | 3,482758621 | |  |

**TABLE 2 – Coronary Flow Reserve and Index of Microcirculatory Resistance parameters – baseline.**

Cells – 0 = Control animals, 1 = MSC animals.

Dp – distal pressure; Tm – mean transit time (1/3 = measure 1, 2/3 = measure 2, 1/3 = measure 3); Hy – hyperemia (after papaverine)

| Cells | Animal | 5minutes  Dp | 5minutes Pd/Tm | 5minutes Dp - Hy | | 5minutes Tm- Hy 1/3 | | 5minutes Tm- Hy 2/3 | | 5minutes Tm- Hy 3/3 | | 5minutes IMR | | 5minutes CRF | |
| --- | --- | --- | --- | --- | --- | --- | --- | --- | --- | --- | --- | --- | --- | --- | --- |
| 1 | 1 | 60 | 32 | 57 | 0,48 | | 0,38 | | 0,48 | | 26 | | 2,48 | |  |
| 0 | 2 | 53 | 30 | 45 | 0,2 | | 0,21 | | 0,23 | | 9 | | 2,5 | |  |
| 1 | 3 | 53 | 62 | 42 | 0,54 | | 0,46 | | 0,57 | | 22 | | 2,25477707 | |  |
| 0 | 4 | 60 | 71 | 47 | 0,36 | | 0,39 | | 0,34 | | 17 | | 3,256880734 | |  |
| 0 | 5 | 79 | 72 | 54 | 0,24 | | 0,12 | | 0,17 | | 10 | | 5,188679245 | |  |
| 1 | 6 | 58 | 19 | 47 | 0,19 | | 0,19 | | 0,12 | | 8 | | 1,9 | |  |
| 0 | 7 | 68 | 32 | 48 | 0,13 | | 0,12 | | 0,12 | | 6 | | 3,864864865 | |  |
| 0 | 8 | 52 | 23 | 43 | 0,19 | | 0,19 | | 0,2 | | 8 | | 2,155172414 | |  |
| 1 | 9 | 50 | 8 | 31 | 0,82 | | 0,79 | | 0,88 | | 26 | | 0,18875502 | |  |
| 1 | 10 | 66 | 34 | 56 | 0,27 | | 0,22 | | 0,28 | | 14 | | 1,974025974 | |  |
| 1 | 11 | 75 | 36 | 65 | 0,16 | | 0,18 | | 0,16 | | 11 | | 2,86 | |  |
| 1 | 12 | 53 | 19 | 37 | 0,51 | | 0,48 | | 0,58 | | 20 | | 0,668789809 | |  |
| 1 | 13 | 53 | 36 | 42 | 0,12 | | 0,12 | | 0,12 | | 5 | | 5,638888889 | |  |
| 0 | 14 | 68 | 34 | 66 | 0,17 | | 0,16 | | 0,12 | | 10 | | 3,377777778 | |  |
| 0 | 15 | 56 | 45 | 36 | 0,29 | | 0,21 | | 0,22 | | 8 | | 3,319444444 | |  |
| 1 | 16 | 69 | 41 | 43 | 0,13 | | 0,14 | | 0,14 | | 6 | | 4,390243902 | |  |
| 0 | 17 | 81 | 45 | 75 | 0,15 | | 0,19 | | 0,15 | | 13 | | 3,408163265 | |  |
| 0 | 18 | 70 | 53 | 45 | 0,16 | | 0,16 | | 0,13 | | 7 | | 4,977777778 | |  |

**TABLE 3 – Coronary Flow Reserve and Index of Microcirculatory Resistance parameters – 5 minutes.**

Cells – 0 = Control animals, 1 = MSC animals.

Dp – distal pressure; Tm – mean transit time (1/3 = measure 1, 2/3 = measure 2, 1/3 = measure 3); Hy – hyperemia (after papaverine)

| Cells | Animal | 5minutes  Dp | 30minutes Pd/Tm | 30minutes Dp - Hy | | 30minutes Tm- Hy 1/3 | | 30minutes Tm- Hy 2/3 | | 30minutes Tm- Hy 3/3 | | 30minutes CRF | | 30minutes IMR | |
| --- | --- | --- | --- | --- | --- | --- | --- | --- | --- | --- | --- | --- | --- | --- | --- |
| 1 | 1 | 46 | 36 | 46 | 0,45 | | 0,53 | | 0,52 | | 2,3 | | 23 | |  |
| 0 | 2 | 61 | 30 | 36 | 0,24 | | 0,12 | | 0,13 | | 3,102040816 | | 6 | |  |
| 1 | 3 | 59 | 59 | 41 | 0,48 | | 0,42 | | 0,44 | | 2,246268657 | | 19 | |  |
| 0 | 4 | 50 | 66 | 38 | 0,35 | | 0,27 | | 0,37 | | 3,545454545 | | 13 | |  |
| 0 | 5 | 71 | 71 | 50 | 0,16 | | 0,21 | | 0,14 | | 5,960784314 | | 9 | |  |
| 1 | 6 | 65 | 34 | 60 | 0,33 | | 0,31 | | 0,25 | | 1,752808989 | | 17 | |  |
| 0 | 7 | 65 | 29 | 54 | 0,11 | | 1,3 | | 0,2 | | 0,826086957 | | 6 | |  |
| 0 | 8 | 63 | 43 | 44 | 0,15 | | 0,16 | | 0,13 | | 4,954545455 | | 7 | |  |
| 1 | 9 | 41 | 18 | 58 | 0,57 | | 0,67 | | 0,51 | | 0,742857143 | | 17 | |  |
| 1 | 10 | 68 | 24 | 57 | 0,18 | | 0,15 | | 0,16 | | 2,142857143 | | 10 | |  |
| 1 | 11 | 78 | 30 | 66 | 0,26 | | 0,23 | | 0,2 | | 1,724637681 | | 15 | |  |
| 1 | 12 | 63 | 34 | 38 | 0,34 | | 0,29 | | 0,37 | | 1,62 | | 13 | |  |
| 1 | 13 | 55 | 24 | 40 | 0,14 | | 0,22 | | 0,16 | | 2,538461538 | | 7 | |  |
| 0 | 14 | 73 | 36 | 72 | 0,17 | | 0,16 | | 0,15 | | 3,083333333 | | 12 | |  |
| 0 | 15 | 57 | 68 | 35 | 0,22 | | 0,26 | | 0,2 | | 5,264705882 | | 8 | |  |
| 1 | 16 | 78 | 62 | 52 | 0,14 | | 0,11 | | 0,16 | | 5,853658537 | | 7 | |  |
| 0 | 17 | 88 | 40 | 83 | 0,14 | | 0,13 | | 0,17 | | 3,159090909 | | 12 | |  |
| 0 | 18 | 51 | 39 | 34 | 0,15 | | 0,19 | | 0,18 | | 4,461538462 | | 6 | |  |

**TABLE 4 – Coronary Flow Reserve and Index of Microcirculatory Resistance parameters – 30 minutes.**

Cells – 0 = Control animals, 1 = MSC animals.

Dp – distal pressure; Tm – mean transit time (1/3 = measure 1, 2/3 = measure 2, 1/3 = measure 3); Hy – hyperemia (after papaverine)
